# MRI-Based Evaluation of Lumbar Epidural Space Depth and Its Correlation with Anthropometric Factors in Saudi Adults

**DOI:** 10.3390/tomography12040053

**Published:** 2026-04-08

**Authors:** Ilhaam Alsaati, Khaleel Alyahya, Mohammed Alharbi, Zuhal Y. Hamd, Shaden Alhegail

**Affiliations:** 1 Department of Basic Sciences, College of Medicine, Princess Nourah bint Abdulrahman University, P.O. Box 84428, Riyadh 11671, Saudi Arabia; 2 Department of Anatomy, King Saud University, Riyadh 11451, Saudi Arabia; 3 Department of Radiology, King Abdullah bin Abdulaziz University Hospital, Riyadh 11671, Saudi Arabiasmalhegail@kaauh.edu.sa (S.A.); 4 Department of Radiological Sciences, College of Health and Rehabilitation Sciences, Princess Nourah bint Abdulrahman University, P.O. Box 84428, Riyadh 11671, Saudi Arabia

**Keywords:** MRI, lumbar, epidural space, Saudi Arabia, L3–L4, L4–L5

## Abstract

**Background**: Epidural procedures benefit from a pre-procedural informed estimation of epidural depth, as anticipating the approximate distance can support safer needle placement and reduce technical difficulties during analgesia or anesthesia procedures. The influence of ethnicity has been established across different populations worldwide; however, there is a lack of Saudi-specific MRI data on epidural depth among the adult population. **Aim of this Study**: To measure the skin to epidural space distance (SED) at the lumbar interspaces L3–L4 and L4–L5 in the Saudi adult population using magnetic resonance imaging (MRI) and to examine its correlations with age, sex, height, weight, and body mass index (BMI). **Methods**: In this retrospective cross-sectional study, sagittal T1-weighted lumbar MRI images of the spine of 169 adult Saudi patients were studied. The age group ranged from 20 to 70 years, with an equal distribution of males and females. The skin to epidural space distance (SED) was measured at the L3–L4 and L4–L5 interspaces, and its correlations with age, sex, height, weight, and BMI were analyzed. **Results**: The average measurement of skin to epidural space distance (SED) was 59.08 mm in L3–L4, and 63.21 in L4–L5. BMI and weight showed strong positive correlations with SED across both levels. Female sex was associated with longer SED values at L4–L5. There was no significant correlation between SED and age or height of the patients. **Conclusions**: MRI-based assessment of SED revealed strong correlations with weight and BMI, but no correlation with height, age, and sex. These findings support the individualized estimation of epidural depth and needle length selection to enhance procedural safety and reduce complications.

## Simple Summary

Epidural anaesthesia necessitates precise assessment of the depth of the epidural space beneath the skin to ensure safe and successful needle insertion. This study employed MRI scans to assess the distance from the skin to the epidural space at two prevalent lumbar levels (L3–L4 and L4–L5) in Saudi individuals and investigated the correlation of this distance with body features. The epidural space was seen to be deeper at L4–L5 compared to L3–L4, with a significant correlation between body weight and body mass index (BMI) and increased epidural depth. Conversely, age, height, and sex revealed no significant impact. These findings underscore the need to take into consideration body weight and BMI when planning epidural treatments in Saudi adults, guiding clinicians in selecting the right needle length and enhancing procedural safety.

## 1. Introduction

Enhancing the effectiveness of any invasive medical procedure requires a thorough and clear understanding of the relevant anatomy. The epidural space is well documented in anatomy textbooks and is an important anatomical region with profound clinical applications in anesthesia practice. Accurate identification of epidural space is crucial for functionality and safety of epidural procedures, as the success percentage of epidural anesthesia varies between 53 and 87% on first attempt [1].

Because epidural depth is strongly influenced by anthropometric and ethnic factors such as BMI, body fat distribution, and vertebral morphology, it is essential for each region or ethnic group to establish population-specific reference values [2,3]. The term “Saudi population” in this study reflects the regional anthropometric profile characteristic of adults living in Saudi Arabia, where the prevalence of overweight and obesity is among the highest globally. These characteristics differ markedly from those reported in East Asian, South Asian, and some African populations, in which lower BMI and different truncal fat patterns have been associated with shallower epidural depths. Given the shared genetic background, lifestyle patterns, and body habitus across the Gulf Cooperation Council (GCC) countries, our findings may also be applicable to neighboring populations in the Arabian Peninsula such as Kuwait, UAE, Qatar, Bahrain, and Oman. However, broader generalization beyond this regional group should be made with caution.

Lumbar epidural techniques are frequently employed in modern medical settings for both surgical anesthesia and pain management. These techniques are often preferred in patients at higher risk from general anesthesia, such as those with cardiopulmonary comorbidities, obesity, advanced age, or anticipated difficult airway—because epidural anesthesia avoids airway manipulation and reduces hemodynamic and respiratory complications. While generally safe when performed correctly, it does carry some associated risks [2], which can arise from two distinct mechanisms. The first includes technical complications related to needle advancement, which are directly influenced by inaccurate estimation of the epidural depth. Inadequate advancement may lead to a false loss of resistance at the ligamentum flavum and subsequent block failure, whereas excessive advancement may result in unintended dural puncture, post-dural puncture headache, or subdural placement. These issues highlight the importance of anticipating the approximate epidural depth before insertion. The second mechanism includes complications related to catheter placement or drug administration—such as infection, epidural hematoma, urinary retention, or inadvertent intravascular or intrathecal injections which are not related to the skin to epidural space distance. These events depend more on sterility, vascular anatomy, and practices other than needle depth. Furthermore, multiple attempts and challenges in accessing epidural space may result in patient pain and discomfort. That’s why ensuring precise needle placement and predicting the preprocedural epidural depth is crucial [4,5]. Epidural depth in the current study refers to skin to epidural space distance (SED). This distance can vary significantly among individuals due to anatomical factors (demographic and anthropometric factors), physiological factors (age and pregnancy), and intervertebral level. The influence of ethnicity on various morphometric SED measurements has been previously established. Ref. [6] mentioned in her review that the greatest SED was found in patients of African origin, and the lowest was found in Chinese parturients. Variations in the average body composition parameters, vertebral anatomy, and soft tissue distribution might explain the SED differences among populations [6]. Imaging technologies such as ultrasound (US), Computed Tomography (CT), and Magnetic Resonance Imaging (MRI) were used to evaluate the SED and correlate it with the actual needle depth to the epidural space. However, while US mostly underestimated SED and CT overestimated it by a few millimeters, MRI proved to be the most accurate and was better at clearly depicting the epidural anatomy [6]. Correlating lumbar SED with patient demographic and anthropometric data has been a consistent theme in the literature worldwide, revealing that certain parameters exhibit stronger predictive values than others. Across all studies, BMI and weight emerged repeatedly as prominent predictors and were strongly positively correlated with lumbar SED, while correlations with age, sex, and height yielded conflicting results. This inconsistency reinforces the need for each population to have its own measurements and correlations.

Epidural anesthesia is a cornerstone of anesthetic practice in Saudi Arabia, widely employed for labor analgesia as well as surgical and perioperative care. It remains the most used form of regional anesthesia for obstetric pain relief [7] and is increasingly applied in surgical populations, including the growing geriatric demographic, where regional techniques are preferred for their safety and opioid-sparing benefits [8]. Despite accumulating studies from multiple populations, MRI-based data specific to Saudi adults are lacking. It is worth noting here that the prevalence of obesity in Saudi Arabia has dramatically increased during the last three decades and become among the highest in the world. Saudi Arabia underwent rapid socioeconomic development, accompanied by substantial lifestyle and environmental shifts that have contributed to rising obesity rates. Urbanization and modernization have led to reduced daily physical activity, with increasing reliance on motorized transport and more sedentary occupations. Parallel dietary changes—characterized by greater intake of calorie-dense foods, refined carbohydrates, fast food, and sugar-sweetened beverages—have replaced traditional nutrient-balanced diets. Additionally, limited opportunities for outdoor physical activity due to climatic conditions, cultural barriers, and low participation in structured exercise—particularly among women—have further exacerbated weight gain. These factors, together with a high national burden of metabolic syndrome and type 2 diabetes, have resulted in a sustained increase in average BMI levels across the adult population. This escalating prevalence of overweight and obesity may influence soft-tissue deposition in the lumbar region and, consequently, epidural depth measurements in Saudi patients [9]. It is reasonable to hypothesize that standard reference depths derived from non-Saudi cohorts may not be directly applicable to local clinical practice. The measurement of SED has direct clinical implications for selecting the appropriate Tuohy needle length. Standard epidural needles measure approximately 8 cm and are adequate for patients with typical SED values of 4–6 cm. For obese patients, in whom SED commonly ranges between 6–8 cm, a longer 9–10 cm Tuohy needle is recommended, whereas in morbidly obese individuals with SED exceeding 8 cm, extra-long needles (11–12 cm) may be required. Anticipating these variations using MRI-based reference values helps anesthesiologists choose the correct needle length in advance, reduce technical difficulty, and minimize the risk of failed blocks or unintended dural puncture. Establishing such anatomical benchmarks is therefore essential to ensure safer and more effective epidural procedures, ultimately improving patient outcomes and satisfaction.

This retrospective cross-sectional study aims to measure the SED at two lumbar levels (L3–L4, L4–L5) using MRI images from a sample of the adult Saudi population and to investigate the correlation of these measurements with age, sex, height, weight, and body mass index. It is also important to note that the present study includes only non-pregnant adults, and the findings are not intended to represent obstetric populations.

## 2. Methods

### 2.1. Study Design and Sampling

This retrospective cross-sectional study was conducted at the Radiology Department of King Abdullah bin Abdulaziz University Hospital in Riyadh, Saudi Arabia. After Institutional Review Board (IRB Log Number: 24-0107) approval was secured, the study analyzed sagittal T1-weighted MR images of the spine of 169 Saudi adults who underwent lumbar spine MRI between January 2022 and April 2024 as outpatients, inpatients, or in the Emergency department. This study sample size calculation is based on the web sample calculator, which used the following equation: Sample size = (Z^2 × P(1 − P))/e^2 ÷ (1 + (Z^2 × P(1 − P)))/(e^2 N), where N = population size, e = Margin of error (percentage in decimal form), z = z-score. The z-score is the number of standard deviations a given proportion is away from the mean. For this study, the confidence level is 95% and the z-score will be 1.96. The age of the participants ranged from 20 to 70 years, with an equal distribution of males and females. With regards to BMI, it was classified according to the World Health Organization categories: underweight (<18.5 kg/m^2^), normal weight (18.5–24.9 kg/m^2^), overweight (25.0–29.9 kg/m^2^), obesity class I (30.0–34.9 kg/m^2^), obesity class II (35.0–39.9 kg/m^2^), and obesity class III (≥40 kg/m^2^). Weight, height, and BMI were recorded for each participant.

### 2.2. Data Collection and Instruments

**Figure 1:**
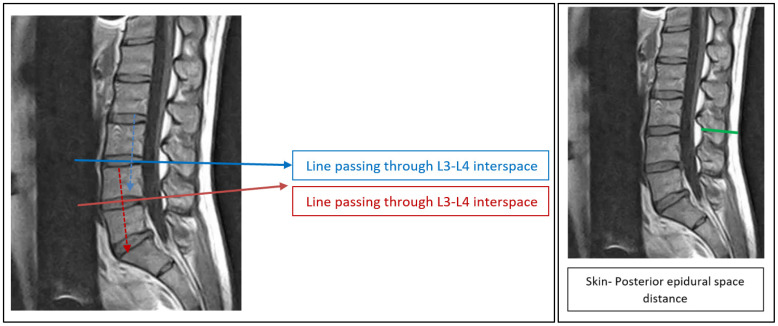
Shows measurement SkinEpidural space.

Patients with MRI evidence of vertebral fractures, herniated discs, masses, tumors, metastasis, vertebral column deformities, congenital anomalies, previous lumbosacral spinal surgery, or poor image quality were excluded from the study. Sagittal T1-weighted images of the lumbar spine were obtained for all patients in the supine position and were analyzed using MRI machines Seimens 3 Tesla Magnetom Vida and MRI Philips 1.5 Tesla Dstream (Achieva). It was all obtained in the supine position because lumbar MRI is routinely performed supine for image quality, immobilization, and patient safety. Although epidural procedures are typically performed in sitting or lateral positions, previous MRI-based studies [10,11,12] have shown that the skin-to-epidural distance remains essentially unchanged between positions, as dorsal soft-tissue thickness does not vary significantly. Therefore, supine MRI was considered an appropriate and validated method for estimating SED. Readings were taken in a dark room for better image viewing; and to avoid eye fatigue, no more than 10 patient MRIs readings were performed in one day. All SED measurements were performed by a single trained investigator. To assess intra-rater repeatability, the investigator repeated each measurement twice on a randomly selected subset of MRI scans, with at least a two-week interval between measurement sessions to minimize recall bias. Intra-rater agreement was evaluated using Bland–Altman analysis and the concordance correlation coefficient (CCC), both of which demonstrated excellent repeatability and no systematic measurement bias. The levels chosen for this study were L3–L4 and L4–L5 interspaces, as they are the most used and clinically relevant sites for lumbar epidural anesthesia. These levels lie safely below the termination of the spinal cord, provide wider interlaminar spaces for easier access, and are frequently adopted in previous studies, allowing consistency and comparability of findings. Before starting the measurements, vertebral count was performed, and the two interspaces were determined. A horizontal line was drawn at each level, where the line passed through the intervertebral discs and was most perpendicular to the long axis of the vertebral bodies and the skin (with the vertebral body axis and epidural space being more perpendicular to the skin). Then, an internal measurement device was used to measure SED at the level of that line, which is defined in this study as the distance from external skin edge to the dural side of ligamentum flavum. In MRI analysis, the SED was measured on a sagittal slice at the level of the intervertebral disc because this plane provides a consistent anatomical reference and is perpendicular to both the vertebral bodies and the epidural space. The horizontal line drawn at each disc level is used purely as a measurement plane and does not represent a needle trajectory; the actual SED was measured perpendicularly from the skin surface to the dural side of the ligamentum flavum. Although the diagram may appear to intersect the spinous processes, this line does not indicate puncture direction but only denotes the axial level for measurement. As MRI is routinely acquired in the supine position, our measurements reflect this standard; previous MRI-based studies have shown that the skin-to-epidural distance remains relatively stable between supine and seated positions, with positional changes primarily affecting interlaminar width rather than dorsal soft-tissue thickness. This method aligns with established MRI-based epidural depth studies in the literature. All measurements were recorded in an Excel sheet for each patient, together with the variables (age, sex, height, weight, and BMI) to be analyzed and correlated.as shown in Figure 1.

### 2.3. Statistical Analysis

Data was analyzed using SPSS version 27 and Jamovi software Version 2.0. Inferential statistics were performed using frequency and percentage for categorical data, followed by descriptive statistics using mean and standard deviation median. Regression analysis and Pearson’s correlation were performed to assess the relationship between the study variables. Furthermore, *t* test and one-way ANOVA were used to assess relations between demographic and width, as it is normally distributed based on Shapiro–Wilk test *p* value > 0.05, in addition to a non-parametric test to assess relation between demographic and other measurements, as they were abnormally distributed based on the Shapiro–Wilk test *p* value < 0.01. For all correlations, a *p* value < 0.05 was considered statistically significant. Because each participant contributed two SED measurements (L3–L4 and L4–L5), we performed level-specific analyses and additionally fitted a linear mixed-effects model to account for within-subject correlation. The mixed model included a random intercept for each participant and fixed effects for vertebral level and the anthropometric predictor (BMI or weight). Model coefficients, 95% confidence intervals, and *p* values were reported.

## 3. Results

The characteristics and demographic findings of the 169 patients included in this study are shown in Table 1. Concerning age distribution, 37.3% were in the age group 36–50 years, followed by age 51–65 years (31.4%), then 20–35 years (21.9%), and over 65 years (9.5%). In terms of sex, 50.3% were females and 49.7% were males. Furthermore, the study demonstrated that about 30.8% were in the normal BMI category, followed by overweight (30.2%) and obesity class I (24.9%). The mean age, height, weight, and BMI of the patients were 46.33 years, 165.82 cm, 77.06 kg, and 28.12 kg/cm^2^, respectively. The study found that the average measurements of SED were 59.08 mm in L3–L4 and 63.21 mm in L4–L5, as shown in Table 2. The results showed that the average L3–L4 SED was slightly greater for females than for males (60.03 mm for females; 58.3 mm for males), as well as for the median SED (57.94 mm in females; 57.1 mm in males). The Mann–Whitney U test results suggested a very small effect size in the difference in L3–L4 SED between females and males (0.03), with females likely having higher values for L3–L4 SED than males; however, the difference was not statistically significant (*p* = 0.714). Although insignificant difference in L4–L5 SED was found in both sexes, females yielded higher mean and median values than males (mean = 65.26 mm in females; 61.22 mm in males: median = 63.03 for females; 61.86 mm for males, *p* = 0.123, r = 0.12, which demonstrated small difference of measurements in both groups), as shown in Table 3. With regards to age, a Kruskal–Wallis test showed that there was no significant difference between the categories of the independent variable age with respect to the measurement of L3–L4 and L4–L5 SED, *p* = 0.254, 0.092, respectively, and Chi^2^ = 4.07, 6.43, respectively. The median measurements were lower in older than in younger age groups. It was 56.23 and 56.65 for more than 65 years and 56.71 and 59.08 for the 20–35 years of age group, in both categories of measurement, respectively, as shown in Table 4. Height of patients at both levels showed no significant correlation with SED. In the regression analysis, a significant impact of weight and BMI on the measurement of SED was observed, with a strong significant positive correlation between weight and SED (*p* < 0.001, r > 0.05). Simple linear regression was performed to quantify the relationship between SED and anthropometric predictors. For L3–L4, BMI was a significant predictor of SED (β = 1.45 mm per BMI unit, 95% CI 1.21–1.69, *p* < 0.001), with the model: SED L3–L4 = 17.62 + 1.45 × BMI. For L4–L5, BMI remained a strong predictor (β = 1.53 mm per BMI unit, 95% CI 1.29–1.78, *p* < 0.001), with the model: SED L4–L5 = 19.11 + 1.53 × BMI. Weight also significantly predicted SED. SED L3–L4 = 19.84 + 0.51 × Weight (kg), β = 0.51 mm/kg (95% CI 0.42–0.59, *p* < 0.001), and SED L4–L5 = 21.07 + 0.52 × Weight (kg), β = 0.52 mm/kg (95% CI 0.44–0.60, *p* < 0.001). These models explain the majority of variance in SED and allow practical, population-specific estimation of needle depth. The Kruskal–Wallis test showed a significant difference between categories of the independent variable BMI with respect to the dependent variable measurement (L3–L4 SED; L4–L5 SED; L3–L4, *p* = <.001, SED increased gradually as BMI increased, (Table 5, Figure 1). The average L3–L4 SED measurements in the underweight, normal weight, overweight, and obese class I, II, and III groups were 43.3, 51.13, 58.31, 63.46, 72.52, and 90.98 mm, respectively. The average L4–L5 SED measurements in the underweight, normal weight, overweight, and obese class I, II, and III groups were 44.59, 54.4, 62.39, 68.84, 77.71, and 93.76 mm, respectively, as shown in Table 5. A non-significant negative correlation was observed between age and median SED at both levels as shown in Table 6.

**Figure 2:**
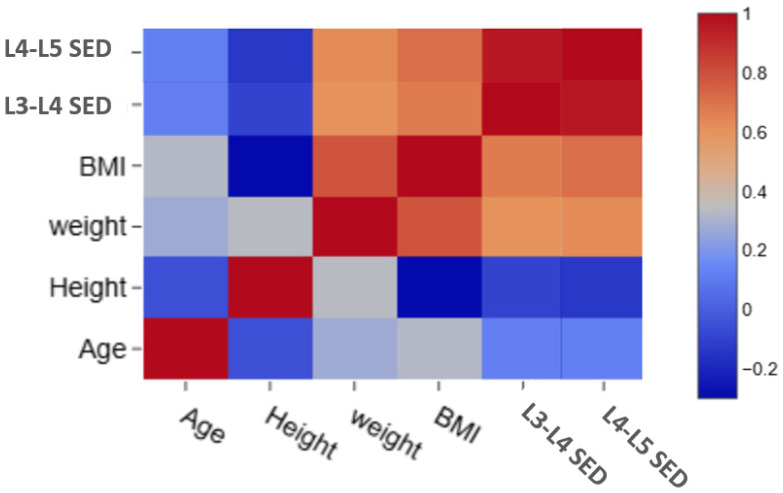
Correlation heat map to assess the relation between demographic character and measurements.

**Figure 3:**
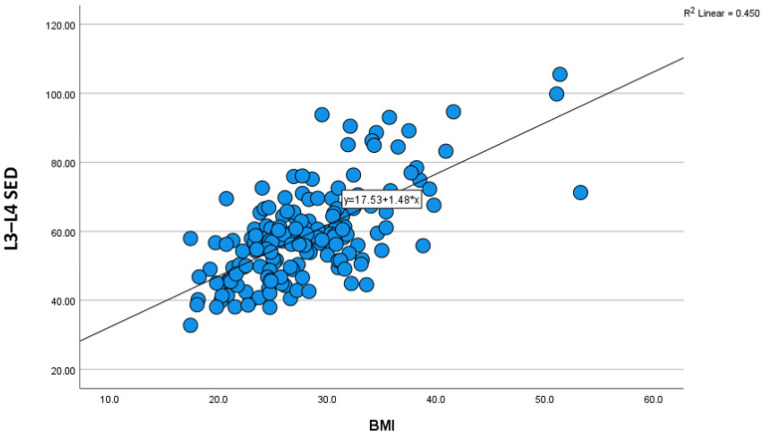
Scatter plot shows linear association between BMI and L3–L4 SED.

**Figure 4:**
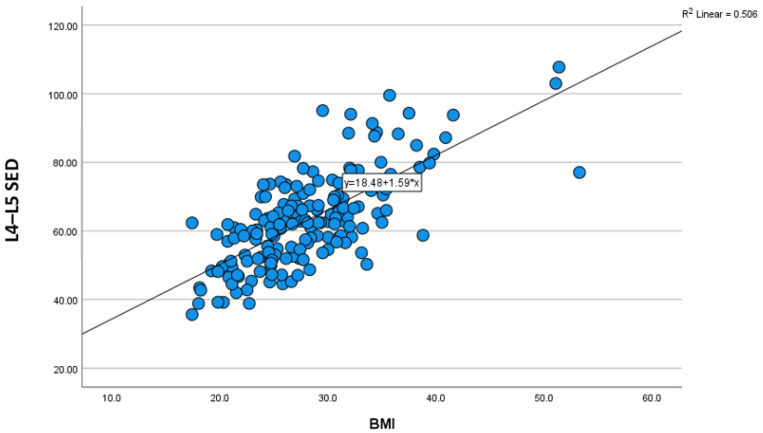
Scatter plot shows strong linear association between BMI and L4–L5 SED.

Intrareader reliability analysis using the Bland–Altman Agreement and CCC shows perfect agreement between the two measurements taken for each category with no bias observed (the mean bias or differences were ≤±0.1) in the Bland–Altman test and a value of CCC range 0.98–0.999, as shown in Table 7. So, measurements are highly reliable and reproducible. We also correlated between demographic characters and measurements as shown in Figure 2, our findings revealed linear correlation between BMI, L3–L4 SED and L4-L5 SED as shown in Figure 3 and Figure 4, respectively.

## 4. Discussion

In this study, we aimed to determine SED at spinal levels L3–L4 and L4–L5 in Saudi adult patients who had previous lumbar MRI, while also examining its correlations with age, sex, height, weight, and BMI. A total of 169 participants were included, with near equal sex distribution (50.3% female, 49.7% male), a mean age and BMI of 46.33 years and 28.12 kg/cm^2^, respectively. This study is representative of middle-aged adults, with a tendency toward overweight/obesity, which is important since BMI strongly influences SED. Our findings indicated that L4–L5 has a slightly greater mean SED than L3–L4, which aligns with the previous literature. Previous studies have been conducted worldwide to establish the correlation between SED and various demographic and anthropometric factors, and ethnic variations were established. This study among Saudi adults reported means of 59.1 mm and 63.2 mm at L3–L4 and L4–L5 levels, respectively. Our values are larger than most Asian or African reports (typically 39–55 mm) [3,10,13,14,15,16,17,18], but approach figures from Western or multi-ethnic obstetric cohorts (≈5–6.5 cm) [19,20,21,22]. This could be due to racial difference, suggesting that Saudis may have more truncal fat distribution than their other populations. The difference in the values affirms the racial influence in the distance from the skin to the epidural space. Alsaaeed et al. [23] as shown in Table 8 concluded that SED in Iraqi obstetric patients in Basrah was greater than that reported in Japanese, Chinese, and Pakistani women but less than that reported in British and German women.

### 4.1. Correlation with Weight and BMI

In the regression analysis, results showed that weight and BMI were strongly positively correlated with SED at both spinal levels. This indicates that as weight and BMI increase, the measurement of SED increases, which is particularly important for anesthetic procedures, as it could affect the success of epidural anesthesia. These results are in agreement with most studies from Japan [11,29], Spain [22], Korea [10], Ireland [21], Iraq [23], India [3,13,15,17], Egypt [25], Greece [26], Indonesia [18,28], Nigeria [14,27], the UK [20], and the US [19]. However, Kaytanci et al. [24] conducted a study in Turkey which concluded that excess weight does not always significantly affect the SED and that the assumption that patients with higher weight will have a SED should be avoided. But unlike our study, they only included individuals with BMI ranging from 18.5 to 29.9 kg/m^2^ and excluded obese individuals (BMI > 30), who are often the most challenging cases for epidural anesthesia, as shown in Table 9.

### 4.2. Correlation with Age

A non-significant negative correlation was observed between age and median SED at both levels as shown in Table 6. Despite this insignificant correlation, the results showed that the SED tends to decrease mildly as patients age. Similarly, Ali and Nosseir [25] demonstrated in their study among Egyptian adults that there is a gradual decline of SED with age [25] and Clinkscales et al. [19] found a modest negative slope among Michigan parturients. Interestingly, Ilori and Djunda in Nigeria [30] and Adachi et al. in Japan [11], inconsistent with this study, found that the mean SED tends to increase with increasing age. Most studies, though, did not find a correlation [3,15,16,17,22,23,27,31]. Agarwal et al. [13] found that age has weak positive correlation with SED in the thoracic region but no correlation in the lumbar region. Another study among a Greek population showed that age was positively correlated with SED in a non-obstetric population, whereas it was negatively correlated in an obstetric population [26].

### 4.3. Correlation with Sex

While most studies found no correlation between sex and SED [3,17,23,27,28] or that it is deeper in males [11,13,15,26,29], our study showed that females exhibited higher mean and median values than males, with this difference being more pronounced at L4–L5, although the difference wasn’t statistically significant. Ilori and Djunda [30] reported a similar sex effect to our study [30] and Ali and Nosseir 2010 found that females had a significantly greater distance than males in the middle-aged population, while males had a greater distance than females in the old-aged population [25]. This could be attributed to the thicker subcutaneous and epidural fat in women and the larger distances reported in parturients [19,23]

### 4.4. Correlation with Height

The correlation between SED and height yielded conflicting results. In our study, no significant correlations with SED were identified at either level. Most studies were consistent with our study and found insignificant correlation between height and SED [3,13,15,22,25,29,31], while other studies contradict this study and found some correlation between SED measurement and height. Bala et al. [16] found that there was an increase in epidural depth with an increase in the height of patients. Conversely, Achi et al., Ilori et al., and Adegboye et al. in their studies among Nigerian adults found negative correlations, suggesting that the taller the individuals the shorter their SED [14,27,30]. Kim et al. [10] in his study in Korea found that height combined with weight had a higher correlation with needle depth than weight or height alone.

All women included in this study were non-pregnant adults undergoing lumbar MRI for clinical indications. Therefore, the SED values reported here reflect a non-obstetric population. Although both pregnancy and obesity can present with elevated BMI, the underlying mechanisms differ in obesity, increased SED is primarily due to greater dorsal subcutaneous fat thickness, whereas pregnancy introduces additional physiological changes—such as elevated intra-abdominal pressure, engorged epidural veins, and alterations in spinal curvature—that affect neuraxial anatomy beyond BMI alone. For this reason, our findings may inform expectations in obese adults but should not be directly extrapolated to pregnant women in labor; interpretation for parturients should rely on obstetric-specific studies. A linear mixed-effects model was used to account for the non-independence of the two SED measurements per participant, and the results were consistent with the level-specific analyses.

Understanding these associations between demographic and anthropometric factors and SED measurements is crucial for optimizing clinical practices, particularly in procedures involving epidural space. To the best of our knowledge, this study represents the first MRI-based evaluation of skin to epidural space distance in the Saudi adult population. While Wani et al. [12] previously reported significant variability in SED measurements across vertebral levels and measurement angles in Saudi children, their findings were confined to the pediatric age group and highlighted the limitations of applying a single predictive formula across all spinal levels. By focusing on adults, the present study addresses an important gap and carries direct clinical implications for anesthetic practice in Saudi hospitals. The larger mean SED values observed in Saudi adults compared with Asian and African cohorts highlight the need for population-specific reference data, as reliance on values from other ethnic groups could underestimate the required distance and increase the risk of multiple puncture attempts, prolonged procedures, or complications. Establishing baseline values for the Saudi population therefore provides anesthesiologists with more accurate expectations of needle depth, enhancing safety, efficiency, and patient comfort during epidural anesthesia. These implications are especially relevant in a population with a high prevalence of overweight and obesity such as Saudi Arabia, as shown in Table 9. Given the strong positive correlation between SED and both weight and BMI, clinicians should anticipate greater technical difficulty in obese patients, select appropriate needle lengths, and consider adjuncts such as ultrasound or MRI-based pre-procedural assessment when needed. Such measures can reduce failed blocks, dural puncture, and vascular injury, ultimately improving procedural safety and patient satisfaction.

## 5. Strengths and Limitations

This study’s strengths include its focus on a specific Saudi adult population, contributing valuable data on anatomical relationships relevant to epidural procedures. However, limitations were present; the study was retrospective, of single-center design and of a small sample size. It excluded non-Saudi patients and individuals with pathological conditions affecting spinal measurements, which may limit the generalizability of the findings. Another limitation is that all patients were studied in the supine position, whereas epidural analgesia and/or catheter placement may be performed in either the sitting or lateral decubitus position. Pregnancy-related anatomical and physiological changes were not assessed in this study, and our SED findings should not be generalized to parturients.

Future multicenter studies are needed to validate these findings and could develop predictive models (e.g., regression models using BMI/weight) or nomograms to assist anesthesiologists in estimating SED for clinical use and also to explore the implications of various spinal pathologies and the effect of position, abdominal girth, and waist circumference on measurement outcomes.

## 6. Conclusions

This study demonstrated that both weight and BMI are significant positive indicators of skin-to-epidural distance (SED), with implications that are both statistically and clinically significant. Age appears to have minimal impact on SED, although there is a slight trend suggesting that older individuals may have shallower epidural spaces. Although females exhibited longer SED than males, this difference was not statistically significant. Height was not significantly correlated with SED. These results support the necessity of tailored preprocedural planning for neuraxial anesthesia, especially among groups with diverse body types and ages. BMI serves as a useful predictor of SED, aiding anesthesiologists in estimating needle depth and minimizing complications. Understanding the impact of these variables can enhance the accuracy and safety of epidural procedures.

## Figures and Tables

**Table 1:** Demographic information of the study participants.

	Demographic Characteristics	Frequency	Percent
**Age**	20–35	37	21.9
	36–50	63	37.3
	51–65	53	31.4
	More than 65	16	9.5
**Sex**	Male	84	49.7
	Female	85	50.3
**BMI**	Underweight	5	3.0
	Normal weight	52	30.8
	Overweight	51	30.2
	Obesity class I	42	24.9
	Obesity class II	14	8.3
	Obesity class III	5	3.0
**Total**	Total	169	100.0

**Table 2:** The mean measurement of age, height, weight, BMI, and MRI measurements.

	Minimum	Maximum	Mean ± Std.	95% CI
**Age**	20	70	46.33 ± 13.66	44.24–48.41
**Height**	117	189	165.82 ± 10.16	164.27–167.37
**Weight**	38.9	153	77.06 ± 16.3	74.57–79.54
**BMI**	17.4	53.3	28.12 ± 6.07	27.2–29.05
**L3–L4 SED**	32.78	105.5	59.08 ± 13.36	57.04–61.11
**L4–L5 SED**	35.63	107.75	63.21 ± 13.56	61.14–65.27

**Table 3:** Comparison between mean SED measurements and sex (Mann–Whitney U test).

Measurements	Sex	Mean	Median	Std. Dev	*p* Value	U	r
**L3–L4 SED**	Female	60.03	57.94	15.38	0.714	3452	0.03
	Male	58.15	57.1	11.07			
**L4–L5 SED**	Female	65.26	63.03	15.07	0.123	3077	0.12
	Male	61.22	61.86	11.66			

**Table 4:** Comparison between mean SED measurements and age group (Kruskal–Wallis test).

Groups	Age	Median	Mean Rank	*p* Value	Chi^2^
**L3–L4 SED**	20–35	56.71	73.66	0.245	4.07
	36–50	59.47	93.49		
	51–65	56.64	82.25		
	More than 65	56.23	86.88		
**L4–L5 SED**	20–35	59.08	69.92	0.092	6.43
	36–50	64.82	95.14		
	51–65	62.03	85.15		
	More than 65	56.65	79.44		

**Table 5:** Comparison between mean SED measurements and BMI groups (Kruskal–Wallis test).

Groups		Mean	Median	Mean Rank	*p* Value	Chi^2^
**L3–L4 SED**	Underweight	43.3	40.21	27.6	<0.001	60.25
	Normal weight	51.13	49.75	53.99		
	Overweight	58.31	58.24	85.84		
	Obesity class I	63.46	60.91	103.7		
	Obesity class II	72.52	72.02	134.29		
	Obesity class III	90.98	94.65	161.2		
**L4–L5 SED**	Underweight	44.59	42.72	21	<0.001	71.48
	Normal weight	54.4	52.67	50.6		
	Overweight	62.39	62.4	85.35		
	Obesity class I	68.84	66.21	108.62		
	Obesity class II	77.71	77.55	136.57		
	Obesity class III	93.76	93.78	160.4		
	Normal weight	20.46	20.7	54.92		
	Overweight	26.79	27.21	84.13		
	Obesity class I	32.54	33.13	106.05		
	Obesity class II	43.02	40.82	138.96		
	Obesity class III	56.69	60.29	158.4		

SED: epidural space distance.

**Table 6:** Correlation between age, height, weight, BMI, and SED measurements.

		L3–L4 PES	L4–L5 PES
**Age**	r	0.117	0.119
	Sig. (2-tailed)	0.128	0.124
**Height**	r	−0.094	−0.136
	Sig. (2-tailed)	0.226	0.078
**Weight**	r	0.604 **	0.624 **
	Sig. (2-tailed)	0.000	0.000
**BMI**	r	0.671 **	0.712 **
	Sig. (2-tailed)	0.000	0.000
**L3–L4 SED**	r	1	0.964 **
	Sig. (2-tailed)		0.000
**L4–L5 SED**	r	0.964 **	1
	Sig. (2-tailed)	0.000	

** means no significant correlation.

**Table 7:** Intrarater reliability analysis for agreement between two measurements for each category using Bland–Altman Agreement and CCC.

Variables	Bland–Altman Agreement	Estimate	Lower C.I.	Upper C.I.	CCC (95% CI)
**L3–L4 Skin SED**	Mean Bias	−0.0900	−0.175	−0.00500	0.999 (0.999–0.999)
	Lower Limit of Agreement	−1.1870	−1.283	−1.09073	
	Upper Limit of Agreement	1.0070	0.911	1.10325	
**L5–L5 Skin SED**	Mean Bias	0.123	0.0338	0.211	0.999 (0.999–0.999)
	Lower Limit of Agreement	−1.023	−1.1239	−0.923	
	Upper Limit of Agreement	1.268	1.1679	1.369	

CCC: concordance correlation coefficient; CI: confidence interval (95%).

**Table 8:** SED values in Saudi Arabia versus international values (in mm) via midline approach.

Study/Population	Sample Size (*n*)	Level Measured	Mean SED (mm)	Tool for Measurements
**Current Study** **Saudi Arabia**	169	L3–L4	59.08	MRI
		L4–L5	63.21	
Kaytanci et al., 2025 [24] **Turkey**	42	L3–L4	43.4	Ultrasound
		L4–L5	45.5	
Agarwal et al., 2024 [13] **India**	194	L4-L5	48	Tuohy needle
Ali and Nosseir 2010 [25] **Egypt**	160	Level of L3 Middle Age Old Age	67 in males and 73 in females 48 in males and 50 in females	Computerized axial scans
		Level of L4 Middle Age Old Age	73 in males and 82 in females 51 in males and 54 in females	
		Level of L5 Middle Age Old Age	64 in males and 71 in females 47 in males and 52 in females	
Adachi et al., 2007 [11] **Japan**	4964	Lumbar levels L1–L2 to L5–S1	41	Tuohy needle
Stamatakis et al., 2005 [26] **Greece**	406	L3–L4 Obstetric females Nonobstetric females	41.4 50.8	Tuohy needle
Clinkscales et al., 2007 [19] **USA**	2009	L3–L4 (Parturients)	54	Tuohy needle
		L4–L5 (Parturients)	54	
Adegboye et al., 2017 [27] **Nigeria**	120	L3–L4 or L4–L5	46	Tuohy needle
Alsaaeed et al., 2018 [23] **Iraq**	255	L3–L4 (Obstetric)	44.3	Tuohy needle
Hartawan et al., 2019 [28] **Indonesia**	56	L3–L4 BMI less than 30 kg/m^2^ BMI more than 30 kg/m^2^	50 60	Tuohy needle
Sharma et al., 2011 [20] **UK**	1210	Lumbar region	54	Tuohy needle
Kim et al., 2023 [10] **Korea**	386	L3–L4	52.17	MRI and C-arm measurement
		L4–L5	52.08	

**Table 9:** Suggested touchy needle length based on BMI categories in Saudi adults.

BMI Category	Typical SED Range *	Recommended Tuohy Needle Length	Clinical Considerations
**Normal (<25 kg/m^2^)**	~4–5 cm	8 cm (standard)	Standard length is usually sufficient; failures are rare.
**Overweight (25–29.9 kg/m^2^)**	~5–6 cm	8 cm (standard)	Adequate in most cases, but caution advised as SED approaches 6 cm.
**Obese (≥30 kg/m^2^)**	~6–8 cm	9–10 cm (long)	Longer needle improves success; anticipate increased difficulty.
**Morbidly obese (≥40 kg/m^2^)**	≥8 cm	11–12 cm (extra-long)	Extended-length needles should be available, especially in obstetric settings.

* Typical SED range represents approximate skin-to-epidural space distance derived from the study findings and related literature; values may vary between individuals depending on anthropometric characteristics.

## Data Availability

The original contributions presented in this study are included in the article. Further inquiries can be directed to the corresponding author(s).
